# Contribution of Eye Movements to Primary-School Children’s Silent Reading Speed: Evidence from Ex-Gaussian Analysis

**DOI:** 10.3390/jemr19040093

**Published:** 2026-08-20

**Authors:** Angeliki Gleni, Sotiris Plainis, Angeliki Mouzaki, Miltiadis K. Tsilimbaris, Panagiota Dimitropoulou, Panagiotis G. Simos

**Affiliations:** 1 Institute of Optics & Vision, School of Medicine, University of Crete, 71003 Heraklion, Crete, Greece; plainis@uoc.gr (S.P.); tsilimb@med.uoc.gr (M.K.T.); 2 Department of Primary Education, University of Crete, 74100 Rethymnon, Crete, Greece; amouzaki@uoc.gr; 3 Department of Psychology, University of Crete, 74100 Rethymnon, Crete, Greece; p.dimitropoulou@uoc.gr; 4 Institute of Computer Science, Foundation for Research and Technology-Hellas (FORTH), 71500 Heraklion, Crete, Greece; simosp@uoc.gr; 5 Department of Psychiatry and Behavioral Sciences, School of Medicine, University of Crete, 71003 Heraklion, Crete, Greece

**Keywords:** eye movements, child reading, silent reading speed, Ex-Gaussian analysis

## Abstract

Reading speed, a core indicator of reading performance, requires efficient oculomotor behavior. While well-documented in adults, the contribution of eye movements to children’s silent reading speed and grade-related changes remain underspecified. Furthermore, the distributional properties of fixation durations have rarely been examined in children. This study investigates how changes in eye-movement parameters associate with individual differences in children’s silent reading speed. The eye movements of 194 typically developing Greek-speaking students (Grades 3–6) were recorded during silent reading of short passages. An Ex-Gaussian distributional analysis was applied to fixation durations, alongside traditional measures of mean fixation number, fixation duration, and saccade amplitude. Language and cognitive abilities were also evaluated. Silent reading speed increased with grade, driven by fewer and shorter fixations, and longer saccades. After accounting for age, vocabulary and cognitive measures, eye-movement parameters increased the explained variance in silent reading speed to 80% (adjusted R^2^ = 0.800) in the full sample (N = 194). The Ex-Gaussian parameter τ was an independent predictor, distinct from mean fixation duration. Finally, age significantly moderated the association between forward fixations and silent reading speed. These findings indicate that oculomotor behavior is independently linked to children’s silent reading speed across primary school years, as captured both by conventional and distributional oculomotor parameters.

## 1. Introduction

Reading is a complex cognitive task, in which efficient processing of written information is highly dependent on coordinated oculomotor behavior [1,2]. As we read, a well-coordinated sequence of saccades (fast ballistic eye movements) and fixations (intervals of relative gaze stability) enables the extraction and processing of textual information. Consequently, reading speed has long been considered an essential indicator of reading performance [2,3,4,5]. It has also been linked to various aspects of information processing related to both textual characteristics and abilities of the reader [1,6]. Furthermore, silent reading speed can be accurately measured through eye-tracking in clinical settings to assess functional vision across different life stages and optical correction modes [7,8,9].

A substantial body of eye-tracking research has revealed robust associations between eye movements and reading performance in skilled adult reading [4,10], which is highly automatized and characterized by relatively low inter-individual variability in silent reading speed. Comparatively fewer studies have evaluated silent reading in children, which exhibits greater variability, as both oculomotor and linguistic–cognitive abilities are still developing [11,12]. Addressing the extent to which eye-movement behavior contributes to children’s silent reading speed beyond established cognitive and language factors remains underspecified. Addressing this question is of considerable theoretical and practical importance, since eye-movement measures can potentially serve as online, objective indicators of reading efficiency [6,13]. Existing evidence suggests that children tend to make more frequent and longer fixations with shorter saccades compared to adults, while fixation durations are approximately 100–150 ms longer [4,10,12,14,15,16], resulting in slower silent reading.

Additionally, relatively few studies have examined the relation between eye movements and silent reading speed across developmental stages [5,17], while even fewer studies have done so in non-English, transparent orthographic systems [14,18,19,20]. Because the large majority of eye-movement research on child reading has been conducted in English, an orthography characterized by comparatively low grapheme–phoneme correspondence, it remains unclear to what extent existing accounts of the relation between oculomotor behavior and silent reading speed generalize to languages with more transparent orthographies. Greek is a highly transparent orthography, with grapheme–phoneme correspondence estimated at approximately 95% for reading, while the majority of students are able to decode multisyllabic words by Grade 3 [21]. These features make Greek a particularly useful test case for examining how oculomotor efficiency during silent reading develops once grapheme–phoneme decoding has been largely established. Yet, eye-tracking studies of Greek-speaking children remain scarce [19,22]. To address these gaps, we examined the oculomotor behavior of typically developing Greek-speaking students attending Grades 3 to 6 in primary school, during silent reading of short, age-appropriate passages for comprehension.

A related, comparatively underexplored issue concerns the characterization of fixation-duration distributions during reading. It has long been established that reading-related fixation durations are not normally distributed [23,24]. Instead, they exhibit a positively skewed distribution because relatively infrequent but substantially longer fixations produce an extended right tail. This pattern is well described by the Ex-Gaussian distribution, a convolution of Gaussian and exponential components that was introduced by Ratcliff (1979) to describe reaction time distributions [25,26]. The Ex-Gaussian distribution is defined by three parameters: μ (mu) and σ (sigma), representing the location and spread of the Gaussian component, respectively, and τ (tau), representing the mean of the exponential component, capturing the rightward skewness of the distribution [25,27,28]. Larger τ values correspond to a heavier right tail, reflecting a higher probability and stronger contribution of prolonged fixations to the overall fixation-duration distribution. The sum of μ and τ equals the mean of the fitted Ex-Gaussian distribution and closely approximates the empirical mean fixation duration [29].

Most eye-tracking studies to date have relied on mean fixation duration as an index of processing time during reading. However, identical changes in mean fixation duration can result from either a shift of the entire distribution toward longer values or the relatively small proportion of unusually prolonged fixations becoming more prominent [25,30]. Analyses based solely on the mean cannot distinguish between these alternatives. Ex-Gaussian analyses address this limitation by decomposing the fixation-duration distribution into components that capture distinct distributional characteristics. Previous studies have shown that different experimental manipulations selectively affect these components [3,23,24,31,32,33]. Stimulus quality, including luminance and text contrast [7,24], lexical ambiguity [33], predictability [27,28], and font characteristics [34], primarily influence the Gaussian component of the distribution. Similar effects have been found under altered visual conditions, such as optical blur, reduced visual acuity, and monocular reading [3,35]. On the other hand, manipulations of task demands, including text length [36] and reading goals (e.g., reading for speed versus reading for comprehension [3]), primarily affect the exponential component. Other factors, such as word frequency [23], parafoveal preview of a word [32], and context, have been shown to impact both components [37]. Collectively, these findings suggest that Ex-Gaussian analyses can distinguish between different sources of variation in reading performance. Although the methodological and interpretational advantages of this framework have been highlighted in adult reading research [33], it has not yet been applied to examine grade differences in children’s oculomotor behavior during text reading.

Accordingly, the current study had three primary objectives: First, we sought to estimate the contribution of eye-movement behavior to individual differences in silent reading speed as typically developing elementary school students enrolled in Grades 3–6 read text passages for comprehension. Specifically, we examined which eye-movement parameters accounted for unique variance in silent reading speed beyond age as well as established linguistic and cognitive factors, including receptive vocabulary, naming efficiency, and general processing speed. Second, we evaluated whether Ex-Gaussian parameters of the fixation-duration distribution provide information beyond conventional oculomotor measures in characterizing individual differences in silent reading speed. We also examined whether age moderated the associations between selected oculomotor measures and silent reading speed. Third, we described grade-related patterns across a broad range of oculomotor indices during passage reading, providing reference values for future studies of children with visual or reading-related difficulties.

## 2. Materials and Methods

### 2.1. Participants

A total of 204 children enrolled in Grades 3–6 were recruited from multiple public primary schools in Heraklion, Crete, Greece. All students attending mainstream classrooms, who were either native Greek speakers or had been enrolled in the Greek public educational system since kindergarten, were invited to participate. Recruitment was independent of reading ability, academic achievement, or teacher referral. Exclusion criteria included ocular or systemic conditions that could affect oculomotor behavior (e.g., amblyopia), spectacle-corrected binocular near visual acuity worse than 0.1 logMAR (decimal acuity < 0.8), clinically significant abnormal phorias, and diagnosis of a specific reading disability or other neurodevelopmental disorder.

### 2.2. Materials and Experimental Procedures

Screening procedures were performed prior to the main experiment to ensure participant eligibility, characterize the sample, and obtain measures that could later be used as covariates in multivariate analyses. First, participants’ binocular near vision was assessed at a viewing distance of 40 cm. Standardized near visual and reading acuity were measured in logMAR units, using the European-wide logMAR chart [37] and the Greek version of the Colenbrander Reading Cards [38], respectively. Assessments took place in a well-lit school room, with chart-background luminance of approximately 70 cd/m^2^.

To verify that participants exhibited age-appropriate language and cognitive abilities and were representative of the typically developing school population, receptive vocabulary was assessed using the LEXiS [39], while oral text reading efficiency and text comprehension were evaluated using the passage reading subtest of the TEST-A battery [40], both standardized assessment tools in Greek. Fluid reasoning and abstract verbal reasoning were evaluated using the Matrices and Similarities subtests of the Wechsler Intelligence Scale for Children—Fifth Edition [41] (Greek edition).

In addition, Rapid Automatized Naming (RAN) digits and objects tasks were administered as measures of naming efficiency and rapid lexical access, while the Symbol Search and Coding subtests of the WISC-V were used as indices of general processing speed, visual scanning efficiency, graphomotor speed, and sustained attention. These latter measures were included as potential covariates in subsequent analyses examining the unique contribution of oculomotor behavior to silent reading speed.

Passage-level silent reading speed and concurrent eye movements were assessed using 5 short passages, purposely composed for the current study. Passages were developed following criteria of the standardized Greek IReST set [42], to ensure appropriateness for primary school children in terms of content and complexity. Passages were also matched for length (mean text length was 75 ± 0.8 words, sentence length was 10.5 ± 0.4 words, word length was 4.05 ± 0.10 letters) and word frequency (mean word frequency was 0.3938 ± 0.0306‰, according to the Hellenic Corpus of the National Thesaurus of the Greek Language [43] (http://hnc.ilsp.gr, accessed on 8 August 2026)). Additional assessment of structural equivalence and readability was implemented via the official Readability Assessment Software (grval 1.1) developed by the Centre for the Greek Language. This tool maps text complexity directly onto the Common European Framework of Reference for Languages (CEFR) levels by utilizing Flesch-adapted metrics for Modern Greek. It was confirmed that all passages fell between A1 and A2 levels, matching the typical reading skills of 7- to 10-year-old primary students and therefore falling well within the expected reading capabilities of the current sample of typically developing children (aged 8–12 years).

To verify that participants read the passages for comprehension rather than merely scanning the text, three closed-ended comprehension questions were administered after each passage. These questions served as a task-compliance check rather than as a separate measure of reading comprehension. Overall comprehension accuracy was thus derived from a total of 15 questions. Responses for one passage were not available for seven participants. To ensure comparability across the sample, comprehension accuracy was expressed as a percentage of the maximum achievable score per child.

During the silent reading task, participants were seated comfortably at a desk in front of the monitor, with their head stabilized by means of a chin rest to minimize head movements. The passages were presented on a high-resolution screen with mean luminance of 50 cd/m^2^, an approximate 100% contrast and a font size of 0.4 logMAR. Binocular recordings of eye movements were carried out using video oculography (EyeLink Portable Duo, SR Research Ltd., Ottawa, ON, Canada). Although the EyeLink Portable Duo supports sampling rates up to 2000 Hz, a sampling rate of 500 Hz was selected following initial pilot sessions, to minimize potential data loss due to technical variability in the experimental conditions (i.e., school environment). A 500 Hz sampling rate provides sufficient accuracy of eye-movement recordings. A standard 5-point calibration/validation procedure was conducted by presenting a small dot (0.3 degrees) on the screen at 0 degrees and at 10 degrees vertically and horizontally, prior to each recording session. The experimental passages were then presented in a pseudo-randomized order across participants. All measurements were completed in a single 45 min session within an appropriately configured room at the participants’ schools.

### 2.3. Data Processing and Eye Movement Measures

Raw eye position data from participants’ right eye were preprocessed and exported using the EyeLink Data Viewer software (version 5.1, SR Research Ltd., Ottawa, ON, Canada). A standard threshold of 70% valid data per passage was applied. A comprehensive dataset of eye-movement and reading measures was subsequently constructed, with further variable computation performed in the R (version 4.5.3) statistical environment.

Silent reading speed expressed in words per minute (wpm) was calculated by dividing the total reading time (from the onset of the first until the offset of the last fixation made on the text area) by the number of text words. A broader set of eye-movement variables was initially examined; the final predictors entered in regression analyses were selected on the basis of theoretical relevance and multicollinearity diagnostics. These included (a) mean fixation duration (measured in milliseconds, ms), (b) number of forward fixations per word (fpw) and regressive fixation frequencies (percentage of backward over total saccades), (c) saccade amplitude (in number of letters) and (d) blink rate (number of blinks per minute, bpm).

Concerning fixation-duration data, a threshold of 80 ms was applied prior to analysis, following standard practice in eye-tracking reading research [44,45,46]. No upper cutoff was applied. This decision was based on exploratory analyses evaluating upper thresholds of 1500 and 1200 ms. Both thresholds affected only a very small proportion of fixations (~0.18%), with no systematic effect on parameter estimates across participants (<5 ms in Ex-Gaussian fitted data). These differences were considered negligible because the overall pattern of results remained unchanged.

Forward fixation durations were fitted with the Ex-Gaussian distribution and parameters μ (mu), τ (tau) and σ (sigma) were estimated in R (version 4.5.2) via the gamlssML() function of the GAMLSS package (version 5.5.0) supplemented by gamlss.dist (version 6.1.1; family = exGAUS()), using maximum-likelihood estimation with a log link for σ and τ and an identity link for μ. Ex-Gaussian models were fitted separately for each passage and subsequently averaged to obtain participant-level estimates. Goodness-of-fit statistics indicated that the Ex-Gaussian model converged successfully for 99.5% of passages, with only 5 passage distributions (0.5%) failing to fit, due to extremely low σ values. Such values indicate that the corresponding distributions deviated from the typical Ex-Gaussian shape observed in the remainder of the dataset. Model fit was assessed by comparing the Bayesian Information Criterion (BIC) of the Ex-Gaussian model against that of a simple Gaussian model fit to the same passage-level data. Model comparison demonstrated that the Ex-Gaussian distribution provided a better fit to the fixation-duration data than a Gaussian-only model in 98.3% of passages, yielding a mean improvement of 54 BIC points. The median BIC of the fitted Ex-Gaussian models was 1373. Grade-specific BIC values for both the Ex-Gaussian and Gaussian-only models are presented in Appendix A, Table A1.

### 2.4. Statistical Analyses

Data was screened for missing values, outliers, and multicollinearity. Extreme multivariate outliers were identified using Mahalanobis distance (*p* < 0.001). Therefore, parametric analyses were considered appropriate for the principal measures, with the exception of blink rate, which was severely non-normally distributed (skewness = 7.67, kurtosis = 67.29). Distributional differences across grade groups were assessed via one-way ANOVAs. For variables showing significant grade effects, independent sample *t*-tests were applied to assess grade differences, supplemented by polynomial trend analyses (linear, quadratic, and cubic contrasts, evaluated at Bonferroni-adjusted *p* < 0.05/6 = 0.008). Blink rate was analyzed via the Kruskal–Wallis test, followed by Dunn’s post hoc tests.

The unique contribution of oculomotor indices to silent reading speed was assessed via hierarchical multiple linear regression analyses, performed in the total sample. Age, gender, receptive vocabulary, naming efficiency (RAN objects and RAN digits), and general processing speed (WISC-V Coding and Symbol Search) were force-entered in the first step. Age was entered in place of grade in regression analyses because, as a continuous variable, it captures developmental variation more precisely across the full age range of the sample. Therefore, although age and grade were highly correlated (*r* = 0.95, *p* < 0.001), the continuous age variable retained more information about within-grade individual differences than the ordinal grade variable. Oral reading fluency was excluded from the primary regression analyses because of substantial conceptual overlap with the dependent variable. All remaining linguistic and cognitive measures demonstrated acceptable collinearity indices.

Eye movement variables were selected from the following list and entered together in the second step: forward fixations per word, mean fixation duration, regression percentage, saccade amplitude, blink frequency, and the three Ex-Gaussian parameters. Because several of these variables exhibited substantial intercorrelations (up to *r* = 0.92), multicollinearity diagnostics were examined before model construction. Multicollinearity was assessed using the Variance Inflation Factor (VIF), adopting a criterion of *VIF* < 3. Skewness and kurtosis ranged between −0.13 and 1.38 and between −1.27 and 3.45, respectively, indicating no substantial deviations from normality. Candidate oculomotor variables were then evaluated with respect to multicollinearity, their bivariate association with silent reading speed, and their relevance to the study objectives. Mean fixation duration and saccade amplitude were excluded due to high VIF values, reflecting substantial collinearity with retained predictors (τ and forward fixations per word, respectively). The Ex-Gaussian parameters μ and σ were excluded due to the absence of a significant bivariate association with silent reading speed. Finally, blink rate was excluded due to the absence of a significant grade-related effect and therefore was unlikely to account for individual differences in reading speed across the sampled age range. Three eye-movement measures—forward fixations per word, regression percentage, and Ex-Gaussian parameter τ—were retained because they showed acceptable VIF values and only moderate intercorrelations (maximum *r* = 0.64) while capturing complementary aspects of oculomotor behavior.

The change in explained variance was used to assess the additional contribution of eye-movement variables beyond that accounted for by age and non-reading-based measures of linguistic and cognitive abilities. Two types of ΔR^2^ are reported: (i) the model-level ΔR^2^, representing the increase in explained variance following the addition of all predictors entered in Step 2, and (ii) predictor-specific ΔR^2^ values (squared semi-partial correlations), representing the unique proportion of variance accounted for by each predictor after controlling for all other variables in the model.

In supplementary models, the potential moderating effects of age on the associations between silent reading speed and oculomotor indices were assessed after mean-centering continuous predictors (evaluated at Bonferroni-adjusted *p* < 0.05/3 = 0.0167). All statistical analyses were conducted using SPSS (version 25).

## 3. Results

### 3.1. Demographics, Visual Function, Language and Cognitive Measures Across Grades

Following initial quality-control procedures, 10 participants were excluded due to incomplete assessment or poor-quality eye-tracking data. Participants were excluded if fewer than 70% of gaze samples were valid in one or more passages, indicating excessive tracking loss or signal noise.

The final sample comprised 194 participants. There were no missing data in the final sample, while four multivariate outliers were detected via Mahalanobis distance (*p* < 0.001). However, the removal of the corresponding cases did not alter the overall pattern of significant findings. Consequently, the results reported are based on the full sample (N = 194).

Grade-level characteristics are reported in Table 1. All participants presented consistently high visual and reading acuity measures across grades, ensuring that they could process the experimental passages without any vision-related difficulties, although differences across grades reached significance in some cases. However, these differences were considered clinically negligible. For instance, the largest observed difference of 0.04 logMAR in visual acuity between Grades 5 and 6 reached statistical significance (*p* < 0.001), although it corresponds to less than half a line in the ETDRS charts. Scores on standardized language, cognitive and oral reading tests indicated that all children performed in the high to low-average range, and grade differences did not exceed *p* < 0.05 on scaled scores or percentile ranks: Participants’ scores on receptive vocabulary (LEXIS) fell within one standard deviation from the normative mean at every grade, while standard scores of oral reading fluency were close to the normative 50th percentile. Participants’ scores on the two processing speed subtests (Coding and Symbol Search) were also within one standard deviation of the normative mean across all grades. Rapid automatized naming (RAN digits and objects) improved by grade, while the two older groups were comparable to each other. Finally, participants demonstrated age-appropriate reading comprehension outcomes (TEST-A comprehension subtest). Age-normed percentile ranks were typical to above average at every grade (Grade 3: 89th percentile; Grade 4: 81st; Grade 5: 67th; Grade 6: 67th), ensuring that the sample exhibited typical reading comprehension abilities. Note that the decrease in percentile scores does not reflect declining comprehension ability, as raw comprehension accuracy in fact increased across the same grade range (Grade 3: 71.5%; Grade 4: 75.6%; Grade 5: 78.5%; Grade 6: 83.1%).

### 3.2. Silent Reading Speed and Eye Movement Measures Across Grades

Descriptive statistics for silent reading speed and eye-movement variables are reported in Table 2, along with performance on comprehension questions as obtained during the silent reading task. Mean values and standard deviations (SDs) presented in this table were calculated based on data aggregated at the participant level.

**Figure 1:**
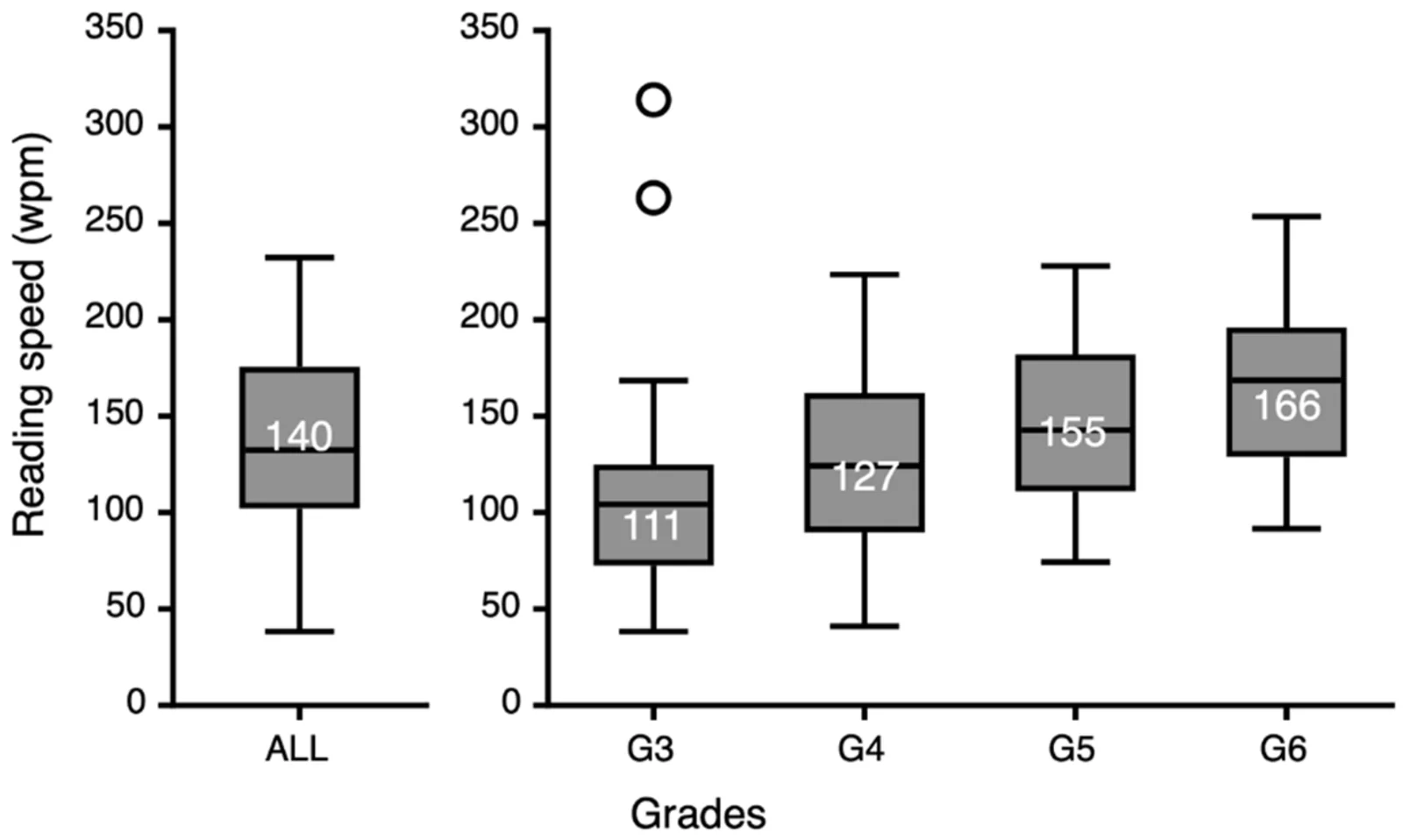
Box plots of silent reading speed (words per minute, wpm) for the total sample (N = 194, **left panel**) and for each grade (G3–G6, **right panel**). Solid horizontal lines within each box represent the median values, numeric labels indicate the mean, and error bars denote ±1 standard deviation.

**Figure 2:**
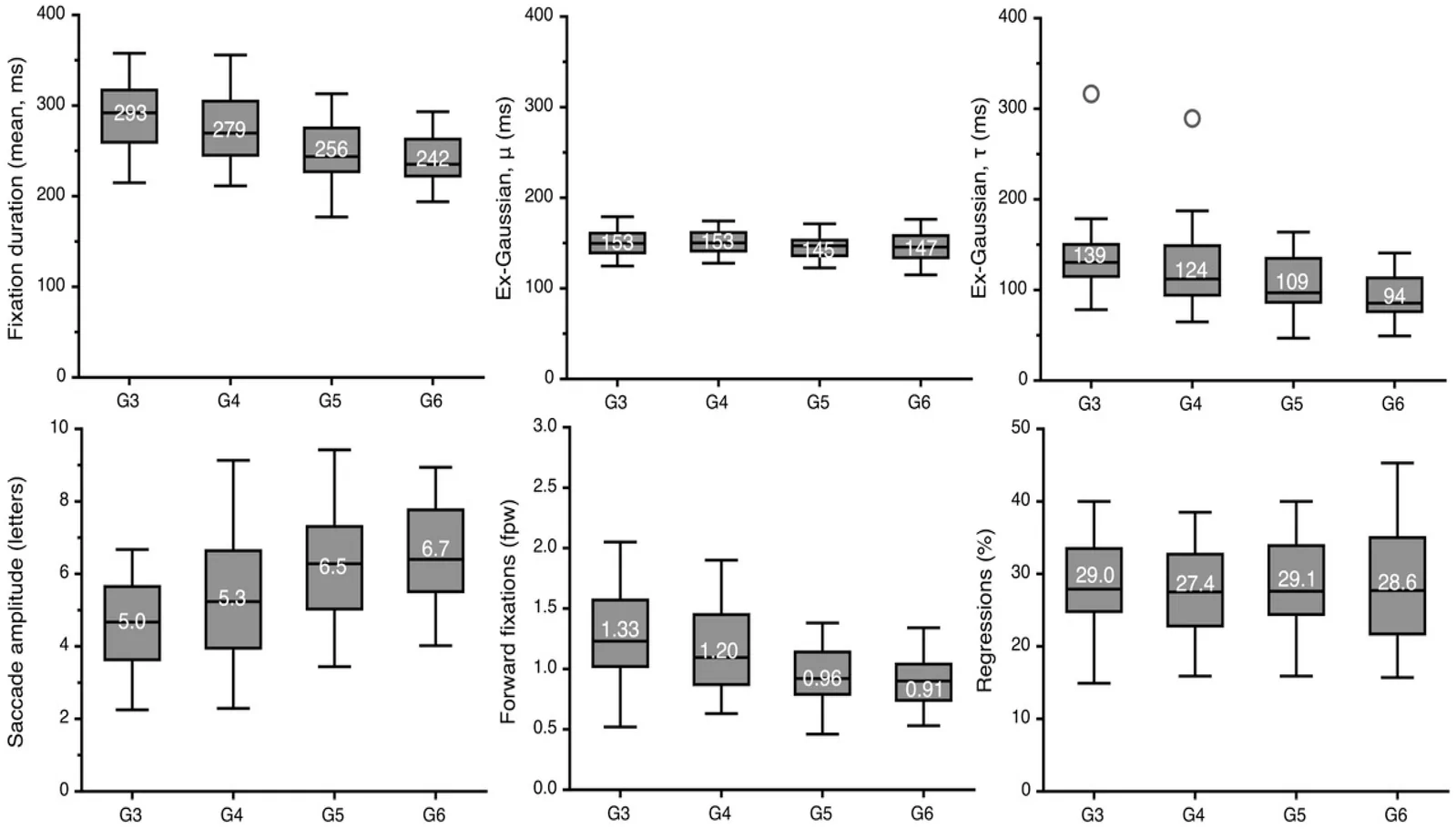
Box plots of eye-movement parameters by grade (G3–G6). The **upper panels** represent fixation-duration parameters (in ms). From left to right, the **lower panels** show saccade amplitude (letters), forward fixations per word and regressive fixation proportion (% of the total fixations). Solid horizontal lines within each box represent the median values, numeric labels indicate the mean, and error bars denote ±1 standard deviation.

One-way ANOVAs showed significant grade-related differences in silent reading speed (*F* (3, 190) = 12.03, *p* < 0.001, *η*^2^ = 0.160), with mean values increasing from 111 (SD = 55) wpm in Grade 3 to 166 (SD = 44) wpm in Grade 6. Polynomial trend analyses revealed a significant linear trend (contrast estimate = 42.98, SE = 7.22, *p* <0.001). Mean saccade amplitude also differed across grades (*F* (3, 190) = 12.54, *p* < 0.001, *η*^2^ = 0.165), with children in lower grades exhibiting shorter saccades (mean amplitude in Grade 3 = 4.9 letters, SD = 1.9) reaching an average amplitude of 6.8 (SD = 1.7) letters in Grade 6 (linear trend = 2.84, SE = 0.478). Similarly, the number of forward fixations per word varied significantly across grades following a linear trend (contrast estimate = −0.338, SE = 0.053, *F* (3, 190) = 14.06, *p* < 0.001, *η*^2^ = 0.182), with mean values below one fixation per word in Grades 5 and 6. Grade-related differences were also observed in mean fixation duration (*F* (3, 190) = 14.54, *p* < 0.001, *η*^2^ = 0.187) and the Ex-Gaussian parameter τ (*F* (3, 190) = 12.83, *p* < 0.001, *η*^2^ = 0.168), both of which showed significant linear trends (contrast estimate = −39.148, SE = 5.949 and −33.697, SE = 5.434, respectively). The quadratic and the cubic trends did not approach significance for any measure (all *p*s > 0.30). In contrast, the proportion of regressive fixations, the Ex-Gaussian parameter μ, and the blink rate did not differ significantly across grades (all *p*s > 0.05). Likewise, the Ex-Gaussian parameter σ did not differ significantly across grades (*F* (3, 190) = 1.56, *p* = 0.200, *η*^2^ = 0.024), with none of the six pairwise comparisons reaching the Bonferroni-adjusted significance threshold (*p* < 0.008). Corresponding box-plots displaying median values are presented separately for silent reading speed (Figure 1) and for eye-movement parameters (Figure 2).

Performance on the comprehension questions indicated a high level of task engagement, with a mean accuracy of 93.0% (SD = 8.0%). Overall, 43.8% of participants answered all questions correctly, and none of the children scored below 50% accuracy. Furthermore, 96.9% achieved at least 75% accuracy, indicating that the vast majority of children were genuinely engaged in reading for comprehension during the eye-tracking task. Although comprehension accuracy tended to be higher in the upper grades, the overall grade effect did not reach statistical significance (Kruskal–Wallis: *H*(3) = 6.81, *p* = 0.078). Exploratory post hoc pairwise comparisons (Dunn’s test) showed lower comprehension in Grade 3 than in Grades 5 and 6 (unadjusted *p* = 0.033 and *p* = 0.021, respectively), whereas no other pairwise comparisons reached significance.

**Figure 3:**
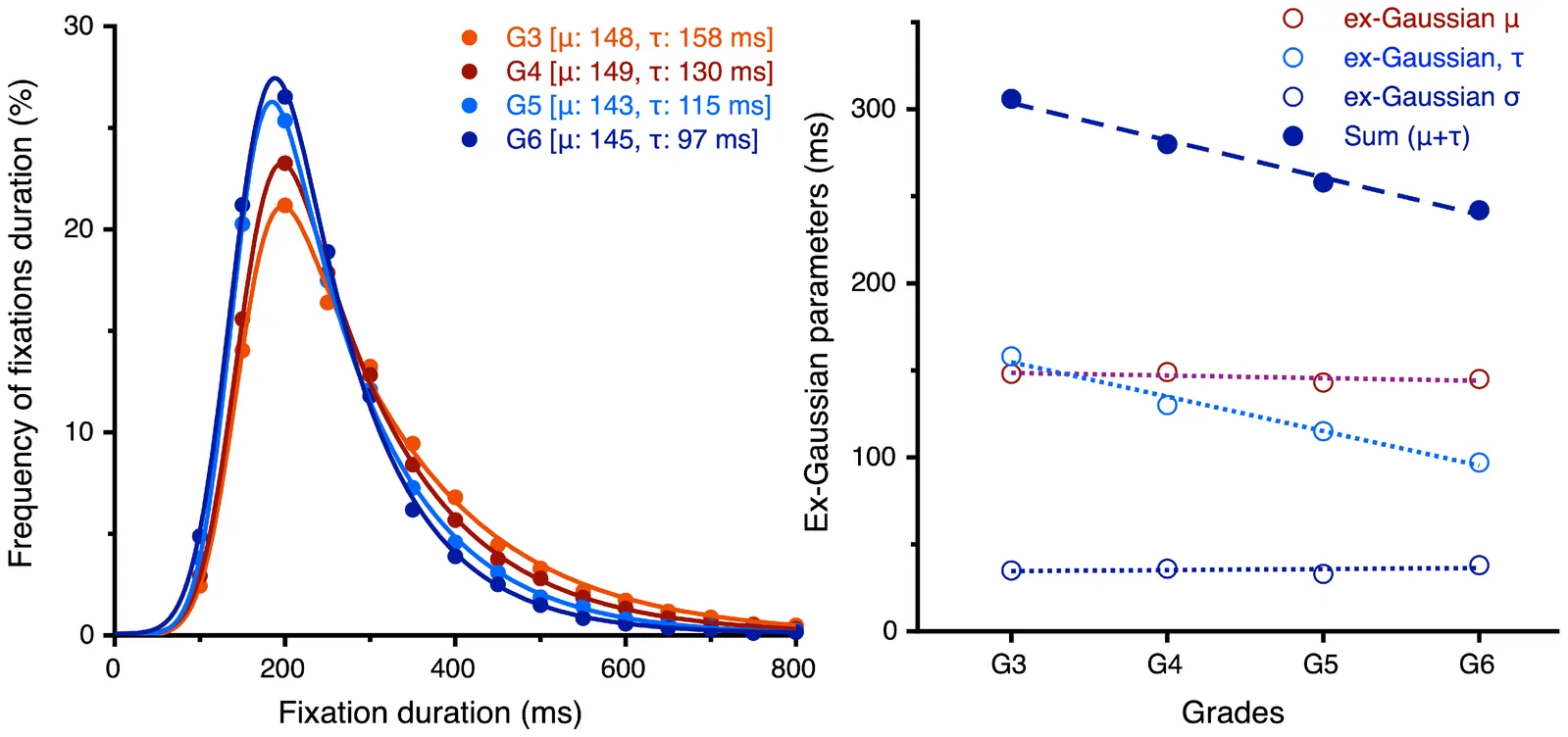
Ex-Gaussian parameter estimates derived from pooled fixation-duration distributions for each grade. Parameter estimates (μ, τ, and σ) were obtained by fitting a single Ex-Gaussian distribution to all forward fixations within each grade (N = 22,910 in Grade 3, N = 20,467 in Grade 4, N = 15,777 in Grade 5 and N = 16,951 in Grade 6). (**Left**) The distributions of fixation durations (50 ms bins) are progressively narrower and with less pronounced right skewness in the higher grades. (**Right**) Grade-specific Ex-Gaussian parameter estimates (μ, σ, τ, and μ + τ, which approximates the mean fixation duration). While μ and σ varied little, τ was progressively smaller in the higher grades, reflecting a reduction in the number of prolonged (>500 ms) fixations.

To further characterize the grade-related differences in fixation-duration distributions, we examined pooled Ex-Gaussian distributions separately for each grade. In addition to the participant-level fitting that was used to obtain individual Ex-Gaussian parameters, fixation durations from all participants and passages were pooled within each grade, and a single Ex-Gaussian distribution was fitted for illustrative purposes (Figure 3). It was evident that the distributions were progressively narrower and less right-skewed in the higher grades. The fitted distributions indicated that the grade-related differences in mean fixation duration were largely attributable to differences in τ, which was progressively lower from Grade 3 (139 ms) to Grade 6 (94 ms; all pairwise comparisons were significant at *p* < 0.001). On the contrary, the μ and σ parameters did not vary significantly across grades.

To assess the extent to which the Ex-Gaussian parameters captured distinct aspects of the fixation-duration distribution, their pairwise correlations were additionally examined within each grade. The parameters μ and σ were strongly and consistently correlated across all four grades (r = 0.60–0.87, *p*s < 0.0001). By contrast, τ showed a negligible-to-weak association with both μ (r = −0.21–0.15, *p*s > 0.10) and σ (r = −0.18–0.18, *p*s > 0.10) across grades.

### 3.3. Bivariate Associations Between Silent Reading Speed and Concurrent Eye Movement Measures

**Figure 4:**
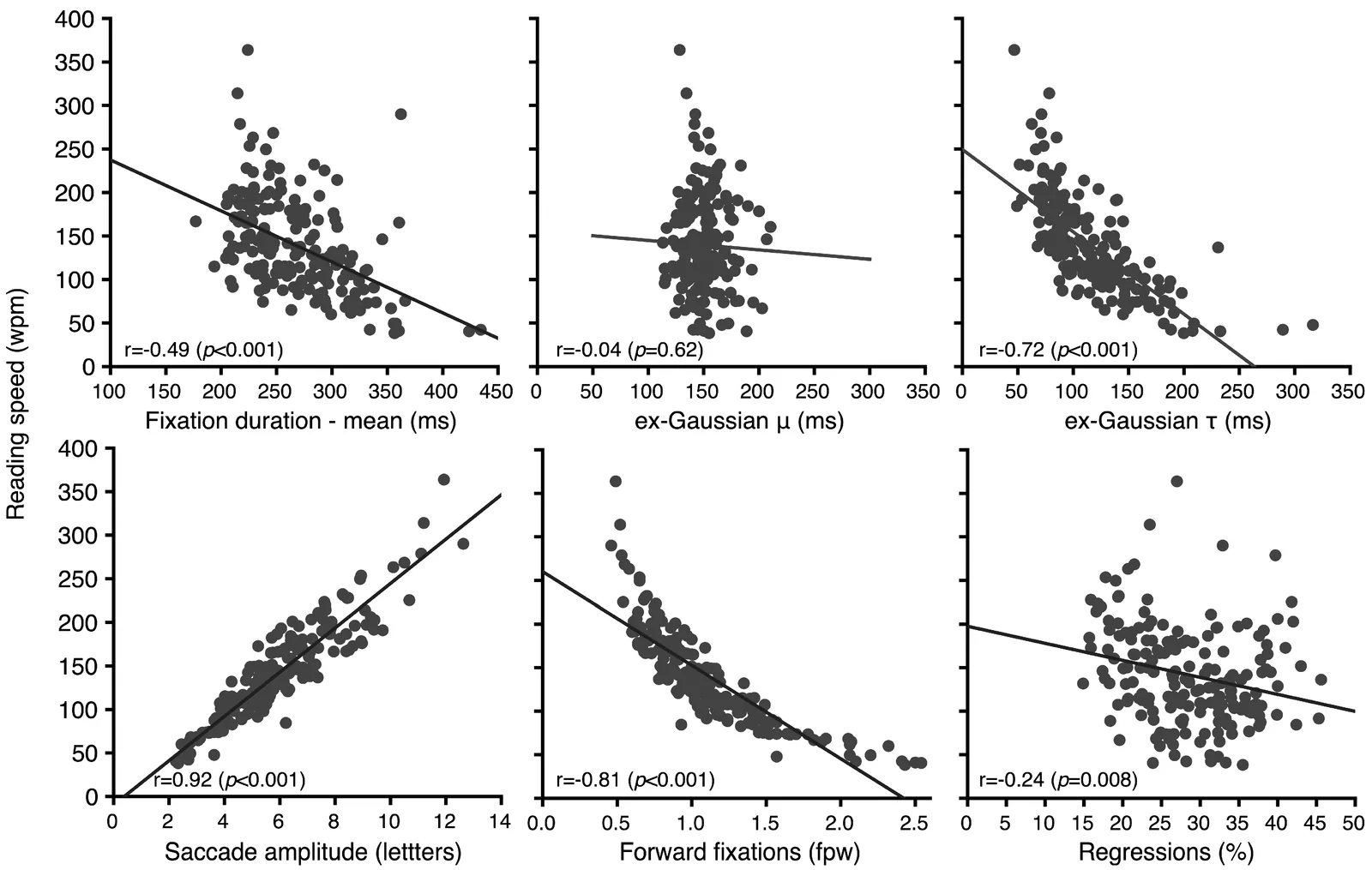
Correlations of silent passage reading speed (wpm) with eye-movement parameters in the total sample (N = 194): Mean fixation duration (**upper left panel**) and the Ex-Gaussian parameters μ (**upper middle panel**) and τ (**upper right panel**); saccade amplitude (**lower left panel**), number of forward fixations per word (**lower middle panel**), and percentage of regressions (**lower right panel**). Correlation coefficients (*r*) and corresponding significance values (*p*) are presented within each panel. Solid lines represent fitted linear trends.

**Figure 5:**
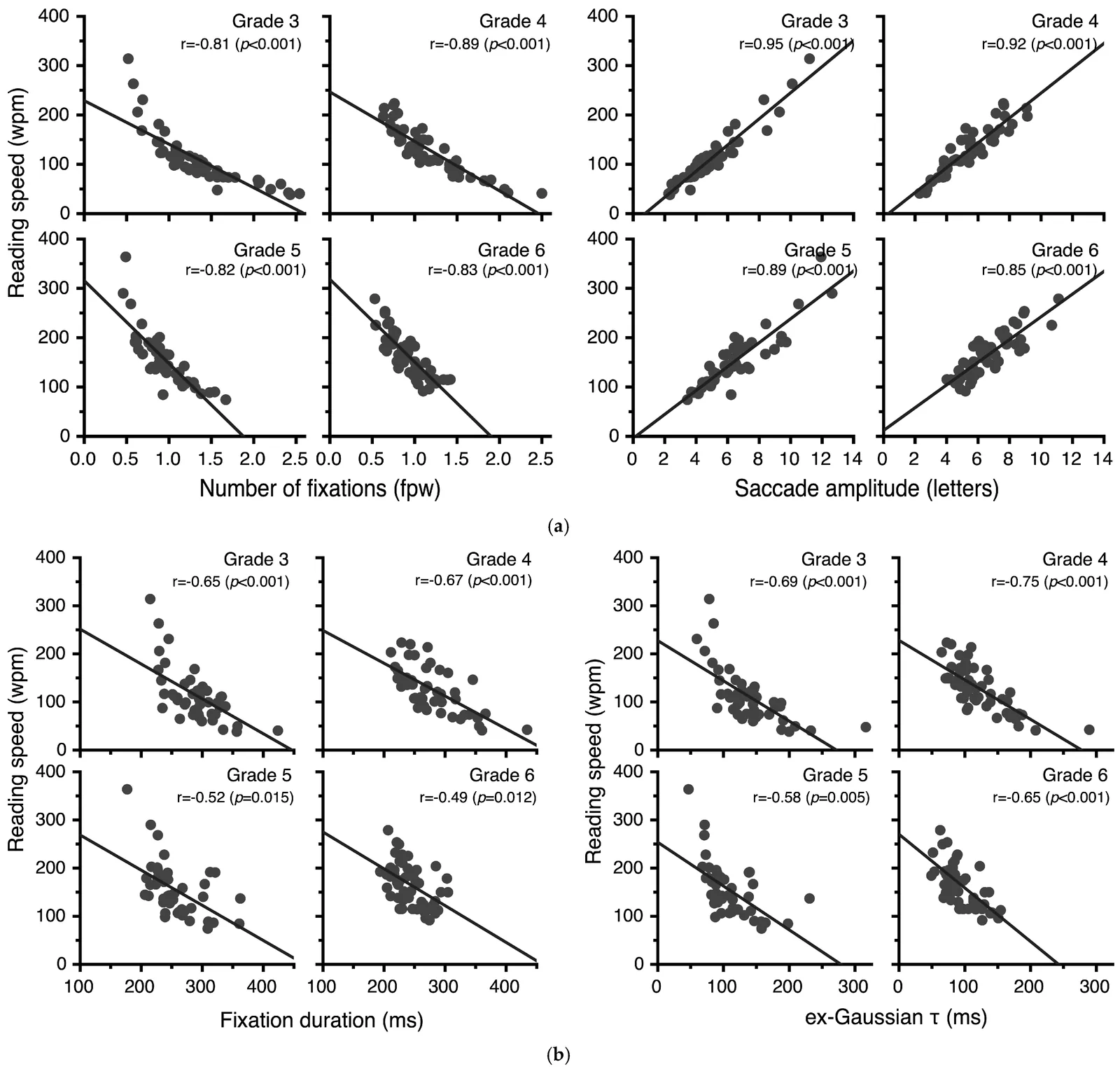
Associations of silent passage reading speed with four key eye-movement parameters per grade. (**a**) Upper panel: number of forward fixations per word (fpw) (**upper left panel**) and saccade amplitude (**upper right panel**). (**b**) Lower panel: mean fixation duration (**lower left panel**), Ex-Gaussian parameter τ (**lower right panel**). Correlation coefficients (*r*) and corresponding significance values (*p*) are displayed within each plot. Solid black lines represent fitted linear trends.

Bivariate correlations confirmed that silent reading speed was systematically associated with core eye-movement parameters (see Figure 4 and Figure 5). In the total sample, the strongest associations were observed with forward saccade amplitude (*r* = 0.92, *p* < 0.001) and number of forward fixations per word (*r* = −0.81, *p* < 0.001), followed by the Ex-Gaussian parameter τ (*r* = −0.72, *p* < 0.001). Modest correlations were found with mean fixation duration (*r* = −0.49, *p* < 0.001), and a weak correlation was observed with regression percentage (*r* = −0.24, *p* = 0.008). The Ex-Gaussian parameters μ and σ did not correlate significantly with silent reading speed (*ps* > 0.05). The blink rate measures were not retained in further analyses, as they did not demonstrate a sufficiently informative contribution during the preliminary evaluation of candidate oculomotor measures.

Figure 5 reveals a similar pattern of associations within each grade group. Forward fixations per word and forward saccade amplitude (Figure 5a), as well as fixation duration—particularly the Ex-Gaussian parameter τ (Figure 5b)—were consistently associated with silent reading speed, whereas regression percentage showed weaker and less consistent associations. The Ex-Gaussian parameter μ was not significantly associated with silent reading speed in any grade (all *p*s > 0.05). Although the strength of some correlations varied modestly across grades, the overall pattern of associations was generally similar across grades. Moreover, age groups differed not only in silent reading speed but also in naming speed and other cognitive and linguistic characteristics associated with reading development. Therefore, moderation analyses are more appropriate to determine whether age alters the unique association between oculomotor behavior and silent reading speed after accounting for concurrent changes in linguistic and cognitive abilities.

### 3.4. Bivariate Associations Between Silent Reading Speed, Language and Cognitive Measures

Language and cognitive measures were also associated with silent reading speed. The strongest correlate was oral reading fluency (*r* = 0.79, *p* < 0.001), followed by RAN Digits (*r* = 0.47, *p* < 0.001). Receptive vocabulary and RAN Objects demonstrated modest associations, whereas Symbol Search and Coding showed relatively weak correlations. Although several associations reached statistical significance within individual grades, the pattern was less consistent than that observed for the principal eye-movement measures.

### 3.5. Independent Predictors of Silent Reading Speed: Multivariate Analyses

A hierarchical regression analysis of the total sample (N = 194) was performed to examine the amount of variance in silent reading speed accounted for by eye movements beyond age, gender, linguistic, and cognitive measures (Table 3). In the baseline model (Table 3, Step 1), age, gender and linguistic–cognitive measures were entered simultaneously in the first step, accounting for 33% of the variance in silent reading speed (Adj. R^2^ = 0.330, F (7, 186) = 14.60, *p* < 0.001). The addition of the three selected eye-movement variables in Step 2 (Table 3, Step 2) resulted in a substantial increase in explained variance (ΔR^2^ (step) = 0.456, Adj. R^2^ = 0.800, ΔF (3, 183) = 147.03, *p* < 0.001). All three eye-movement parameters emerged as significant independent predictors of silent reading speed. The strongest predictor was the number of forward fixations per word (β = −0.622, *p* < 0.001), followed by the Ex-Gaussian parameter τ (β = −0.256, *p* < 0.001) and the percentage of regressions (β = −0.205, *p* < 0.001). Note that individual ΔR^2^ (variable) values (0.200, 0.038, and 0.033) sum to less than ΔR^2^ (step) (0.456), because the predictors share considerable variance (maximum *r* = 0.64), which cannot be uniquely attributed to any single predictor. Following the inclusion of eye-movement variables, age, gender, and all processing speed measures no longer contributed significant unique variance to silent reading speed. Receptive vocabulary was the only non-oculomotor predictor that remained statistically significant, although its unique contribution was comparatively small (β = 0.105, *p* = 0.004). Model diagnostics indicated a modest departure from normality in the residuals (Shapiro–Wilk W = 0.851, *p* < 0.0001) and marginal heteroscedasticity (Breusch–Pagan *p* = 0.023). Given the N (194)/k (10) ratio, coefficient estimates are expected to remain reasonably robust to these modest departures from these assumptions.

To further evaluate the added value of the Ex-Gaussian analysis, a nested-model comparison was conducted between a baseline model including the covariates and mean fixation duration and an extended model that additionally included the Ex-Gaussian τ. The extended model (including τ) provided a significantly better fit (ΔR^2^ ≈ 0.06, *F* (1, 184) = 26.5, *p* < 0.001), with τ remaining a significant unique predictor (*p* < 0.001) while mean fixation duration became non-significant (*p* = 0.55).

To examine the stability of these findings across primary school years, the same regression models were applied separately within each grade (see Table A2, Appendix A). The same three eye-movement variables remained significant predictors across all grades (all *p*s ≤ 0.017). The number of forward fixations per word remained the strongest predictor in every grade (*β* values ranging from −0.631 in Grade 3 to −0.728 in Grade 6). Collectively, the selected eye-movement and linguistic–cognitive predictors accounted for a substantial proportion of variance in silent reading speed, with adjusted R^2^ values ranging from 0.75 (Grade 3) to 0.87 (Grade 6).

### 3.6. Age-Related Moderation of Eye-Movement Predictors of Silent Reading Speed

Finally, we examined whether the degree of the association between each eye-movement parameter and silent reading speed varied significantly with age in the context of four additional multiple linear regression models. In these models, interaction terms between age and each of the three selected eye-movement predictors were included in addition to age and the corresponding eye-movement parameter (mean-centered). Age, gender, RAN Digits and Objects, WISC-V Coding and Symbol Search, and receptive vocabulary raw scores were included as covariates (overall fit statistics for the full moderation model: *R*^2^ = 0.773, *Adj. R*^2^ = 0.761, *F* (9, 184) = 69.46, *p* < 0.001, N = 194).

A significant interaction was observed between age and the average number of forward fixations per word (B = −27.1, SE = 4.9, *p* < 0.001). Examination of simple slopes revealed that the magnitude of the negative association between the number of forward fixations per word and silent reading speed was weakest among children averaging 8.6 years (B = −84.2, SE = 6.3, *p* < 0.001), progressively increased among children averaging 10.1 years (B = −116.6, SE = 6.4, *p* < 0.001), and peaked in the oldest age group, which averaged 11.9 years (B = −159.3, SE = 12.2, *p* < 0.001).

Although the interaction between age and percentage of regression fixations did not reach significance (B = 0.7, SE = 0.4, *p* = 0.09), the inspection of simple slopes indicated that the negative association between percentage of regression fixations and silent reading speed was significant only in the two youngest age groups (B = −2.5, SE = 0.8, *p* = 0.002 and B = −1.7, SE = 0.5, *p* < 0.001; B = −0.7, SE = 0.7, *p* = 0.30 in the oldest age group). Conversely, the magnitude of the association between silent reading speed and the Ex-Gaussian parameter τ did not vary significantly with age (*p* = 0.30).

## 4. Discussion

The present study combined conventional eye-movement measures with Ex-Gaussian distributional analyses to investigate the relationship between oculomotor behavior and silent reading speed in typically developing children reading connected text. The main findings can be summarized as follows: First, oculomotor measures accounted for substantial unique variance in silent reading speed beyond age and established linguistic and cognitive correlates. Second, distributional analyses demonstrated that grade-related differences in fixation duration were primarily attributable to changes in the right tail of the fixation-duration distribution (τ), rather than to a global shift in fixation duration. Third, substantial grade-related differences were observed in several oculomotor measures, and moderation analyses indicated that age influenced the strength of some—but not all—associations between eye movements and silent reading speed. Each of these findings will be discussed in turn below.

### 4.1. Contribution of Eye Movements to Silent Reading Speed

A central finding of the study was the strong association between silent reading speed and oculomotor behavior, while regression analyses identified the eye-movement parameters that accounted for unique variance in silent reading speed, namely the number of forward fixations per word, regression percentage, and the Ex-Gaussian parameter τ, which reflects prolonged fixation duration. More importantly, these measures accounted for a substantial proportion of variance beyond age, naming efficiency, receptive vocabulary, and general processing-speed measures (approximately 46%). Once eye-movement parameters entered the model, age and cognitive variables no longer contributed unique variance.

These findings suggest that oculomotor behavior captures aspects of reading efficiency that are not fully explained by broader linguistic or processing-speed factors. The contribution of the Ex-Gaussian parameter τ to children’s silent reading speed is a novel addition to the eye-movement literature in primary school populations. It provides valuable insights into how the skewness of the right tail of the fixation-duration distribution highlights a unique aspect of silent reading performance. Overall, in line with previous work by Blythe [13], improvement in silent reading speed measures is closely paralleled by concurrent changes in eye-movement characteristics, although the cross-sectional design does not permit conclusions regarding the causal direction of these associations.

Regression percentage emerged as an independent correlate of silent reading speed in the regression analyses; on average, its value was comparable across grade groups. Thus, it appears that children who produced a higher proportion of regressions within their respective age group tended to read more slowly. This pattern suggests that regression percentage captures individual differences in reading efficiency that are not apparent from grade-level differences in its mean value. This finding is consistent with the notion that regressions are an integral component of normal reading rather than simply erroneous eye movements, serving multiple functions, including rereading and resolving difficulties or ambiguities in text processing [47,48].

Receptive vocabulary was the only non-oculomotor predictor that remained significant after inclusion of the eye-movement variables. This finding suggests that vocabulary knowledge contributes to silent reading speed through mechanisms that are partly independent of oculomotor efficiency, potentially reflecting differences in lexical access and language processing [49,50].

Despite the consistently strong associations between eye-movement parameters and silent reading speed, moderation analyses revealed that the association between forward fixations per word and silent reading speed became progressively stronger from Grades 3 to 6. Conversely, the association between regression percentage and silent reading speed was stronger among younger readers. The stable association between the Ex-Gaussian parameter τ and silent reading speed further suggests that, unlike regression percentage, the Ex-Gaussian parameter τ shows a relatively invariant association with silent reading speed across the examined age range, indicating that different oculomotor parameters relate to reading performance in different ways.

### 4.2. Implications of Ex-Gaussian Distributional Analyses

The Ex-Gaussian framework has established the role of distributional measures in adult reading, attempting to differentiate between low-level processing and cognitively demanding oculomotor behavior [23,24,25,51]. However, its application to developing populations remains unexplored. The current study is one of the first to apply Ex-Gaussian distributional analysis to fixation durations of primary school children during the critical early years of reading development, addressing a methodological gap in the reading research. Furthermore, the current analyses provide more sensitive indices of grade-related differences in oculomotor behavior than mean fixation duration, which has traditionally been examined.

Within this framework, distributional analyses of fixation durations made it possible to examine whether grade-related differences in mean fixation duration reflected a uniform reduction in the entire distribution or changes in specific components of the distribution. Fitting Ex-Gaussian distributions to the fixation-duration data and comparing their parameters across grades demonstrated that the progressive reduction in mean fixation duration in the higher grades (293 ms in Grade 3 to 242 ms in Grade 6) was mainly driven by a reduction of longer fixations. Importantly, the Ex-Gaussian parameters exhibited differentiated patterns: the μ parameter, reflecting the central tendency, remained stable across grades (145 ms in Grade 3 to 154 ms in Grade 6), whereas τ, corresponding to the rightward tail, was substantially lower in the higher grades (by almost 45 ms). Thus, older children did not simply show shorter fixations. Instead, they showed fewer unexpectedly long fixations. This distinction would not be apparent from mean fixation duration alone [23,25,52].

The intercorrelations between the Ex-Gaussian parameters are also indicative of their functional distinction. Across grades, a moderate-to-large size association was noted between μ and σ, whereas the magnitude of the association of τ with both μ and σ was very small. These findings are consistent with previous applications of Ex-Gaussian modelling in eye-movement research, suggesting that τ captures a component of fixation-duration variability that is largely independent of the Gaussian parameters and therefore might reflect a distinct aspect of reading behavior [25].

These differentiated patterns of the Ex-Gaussian parameters offer valuable implications for models of eye-movement control during highly complex tasks, such as reading [23,25,51]. The selective reduction in τ in the present study is consistent with the shift from slower, decomposition-based word recognition to faster, more efficient lexical processing as reading skill develops [18]. This is in line with the E-Z Reader framework, within which fixation duration is understood to be regulated by the time required for lexical processing, which depends on visual encoding [53,54]. This interpretation is supported by independent non-oculomotor evidence from the present sample. Performance on Rapid Automatized Naming (RAN), which indexes the speed of phonological and lexical retrieval, improved systematically across grades, paralleled by higher oral reading fluency. The convergence of these patterns with the observed reduction in τ provides evidence that the decrease in prolonged fixations reflects increasingly efficient lexical access rather than a uniform acceleration of all eye movements.

Notably, the visual characteristics of the reading materials were controlled in the current design: print size was well above participants’ visual acuity threshold, and passages were presented in high contrast and high screen luminance conditions. These manipulations reduced the likelihood that grade-related differences in low-level visual characteristics affected the results. The relative stability of μ is thus consistent with previous findings demonstrating changes in this parameter with manipulations of visual quality such as defocus, astigmatic blur, and low contrast [7,8,24]. In contrast, significantly lower τ estimates were found in the upper grades. Given that the same experimental passages were used across grades, their readability was well within the age range of the current sample, and very high comprehension accuracy scores were achieved. It is unlikely that grade differences in τ were caused by visual characteristics of the reading material or failure to engage with the reading task. This finding is consistent with the notion that the Ex-Gaussian parameter τ may reflect sensitivity to higher-order processes, including lexical and contextual processing [3,23,25,33]. However, this interpretation remains tentative, as the present study did not explicitly manipulate these processes.

### 4.3. Changes in Eye Movement Parameters Across Grades

The present cross-sectional data revealed that silent reading speed was higher by approximately 50% in Grade 6 as compared to Grade 3, accompanied by significantly lower forward fixation counts and mean fixation duration as well as progressively higher saccade amplitude. Furthermore, polynomial trend analyses revealed that grade differences showed a linear trend for all variables across the grade range of this study. This pattern aligns with previous work showing that older and more proficient readers execute progressively longer saccades and fewer fixations, likely reflecting greater automatization and increased reading proficiency [2,11,12,14].

Interestingly, the percentage of regressions relative to the total fixations within each passage remained mostly stable across grades, despite a significant reduction in the absolute number of regressive eye movements. In line with developmental eye-movement models [2,47,48], regressive fixations increase proportionally with the number of forward fixations as both reading skill and eye-movement control develop.

The present findings are part of an emerging body of eye-tracking studies on silent reading in Greek, which has, to date, focused primarily on adult and clinical populations [3,7,19,22]. Developmental eye-tracking evidence from a Greek sample of typical and dyslexic readers has similarly reported that oculomotor parameters improve with age, with a shift toward a silent-reading preference emerging around Grade 4 [19]. This pattern is broadly consistent with the progressive changes in oculomotor efficiency observed in the present sample. Moreover, the Ex-Gaussian τ observed in Grade 6 (94 ms) falls within the range reported previously for Greek adults [3], whereas younger children’s values (Grade 3: 139 ms) sit well above it, extending this convergence pattern to the distributional characterization of fixation duration. One of the most interesting findings that is new to this emerging body of research is the finding that even after basic decoding becomes increasingly automated in a transparent orthography (in upper primary school grades), substantial grade-related changes in oculomotor efficiency continue to occur.

Taken together, these findings suggest that age-related differences in silent reading efficiency involve at least two complementary changes in oculomotor behavior. First, children progressively sample larger portions of text with each saccade, resulting in fewer forward fixations. Second, the processing time devoted to individual fixations decreases, largely through a reduction in unusually prolonged fixations rather than a uniform shift of the entire fixation-duration distribution. These complementary changes likely reflect increasing efficiency in both visual sampling and lexical processing during reading.

### 4.4. Cross-Linguistic Implications

The current study contributes to the exploration of cross-linguistic diversity by examining grade-related changes in oculomotor behavior in a language with transparent orthography that is less explored compared to the opaque orthographic system of English. In English, studies have consistently reported progressive reductions in fixation number and fixation duration, accompanied by increases in saccade amplitude, throughout primary school [12]. Similar developmental patterns have also been observed in relatively transparent orthographies, including Italian [20] and Spanish [17], although the absolute magnitude of these changes varies across orthographic systems. In the present Greek sample, silent reading speed increased from approximately 111 words per minute in Grade 3 to 166 words per minute in Grade 6, while mean fixation duration decreased from 293 ms to 242 ms and average saccade amplitude increased from 4.9 to 6.8 letters. These values fall within the range reported for children reading other alphabetic orthographies, supporting the view that increasing oculomotor efficiency is a robust characteristic of reading development, despite differences in orthographic transparency. Although the present study did not directly compare orthographies, these findings are consistent with the view that age-related changes in eye-movement behavior reflect increasing efficiency in lexical access and reading fluency rather than decoding skill alone.

Therefore, the present findings extend the relatively limited body of eye-tracking studies on transparent orthographies [14,18,20], enhancing evidence that eye-movement efficiency constitutes a cross-linguistic property of reading acquisition across alphabetic systems. Nevertheless, direct cross-linguistic studies employing comparable experimental paradigms are required to determine the extent to which oculomotor trajectories differ as a function of orthographic transparency. Because decoding accuracy is acquired relatively early in Greek [21], changes in silent reading speed may provide a clearer view of the contribution of oculomotor efficiency to fluent reading, beyond conventional measures of processing speed.

Finally, this is one of the few studies applying the Ex-Gaussian distribution analysis of fixation durations during reading in a non-English language [51,55]. The finding that the Ex-Gaussian parameter τ explained unique variance in silent reading speed beyond conventional mean fixation duration further demonstrates that the additional information captured by distributional analyses extends to a highly transparent orthography, supporting the broader cross-linguistic applicability of distributional analyses of fixation durations.

### 4.5. Clinical Implications

Silent reading speed could provide a more direct estimate of visual word recognition efficiency than the current gold-standard (oral reading speed), which is partly constrained by articulation (e.g., eye–voice span and prosody) [56,57]. In future research, oculomotor indices could contribute to the differentiation among distinct cognitive phenotypes, especially by comparing clinical populations characterized by prevalent reading comprehension deficits (e.g., developmental language disorder) against those with prevalent decoding deficits (e.g., developmental dyslexia). Furthermore, eye-movement metrics may provide complementary information about the executive control of eye movements and visual-attentional processes, which contribute to poor reading comprehension in children. For example, children with amblyopia have been shown to read at substantially lower speed than their non-amblyopic peers, with binocular reading characterized by increased forward eye movements and fixation instability [58,59]. Similarly, children diagnosed with dyslexia or ADHD exhibit longer fixation durations, increased regressive eye movements and inefficient saccades compared to typical readers [60,61]. Therefore, oculomotor measures associated with silent reading speed may serve as objective markers of reading difficulty, with potential applications in early identification and intervention monitoring.

In addition, the eye-movement parameters that emerged as significant predictors of silent reading speed in the present study provide reference values for typically developing children. Such reference data may serve as useful benchmarks for future studies examining atypical eye-movement behavior in children with visual impairment or neurodevelopmental conditions affecting reading.

### 4.6. Study Limitations

Several limitations should be acknowledged. First, the present study employed a cross-sectional design, comparing children in Grades 3 through 6 at a single time point. Consequently, the observed differences reflect between-group comparisons and should not be interpreted as evidence of within-child developmental change. Due to this limitation, it cannot be ruled out that some of the observed differences across grades derive from exogenous factors (such as the level of exposure to reading materials, classroom practices etc.). Longitudinal studies with Greek-speaking student populations are therefore needed to further examine whether the eye-movement parameters that emerged as significant predictors of silent reading speed in the current setting can also predict individual silent reading speed improvements over time.

Second, because the present sample comprised typically developing children recruited from mainstream schools, variability in functional vision as well as in several cognitive and linguistic measures was narrower than might be expected in more heterogeneous populations. Future research involving more heterogeneous samples, including children with reading difficulties, is needed to determine the extent to which the present findings generalize beyond typically developing readers.

Third, the current study applied a passage-level rather than sentence or word-level analysis. This allows for a more naturalistic measure that reflects reading in educational settings. It also allows for maximum reliability of distributional analyses. However, it remains possible that local textual effects may be obscured. Phrase- and word-level analyses are planned to clarify how such local textual factors contribute to fixation behavior and reading performance. Additionally, the controlled, age-appropriate nature of the passages also may have contributed to consistently high scoring on comprehension questions. Aggregating comprehension accuracy scores across all passages provided a more reliable index of overall engagement with the reading materials, compared to single passage accuracy. Overall, reading comprehension accuracy displayed a ceiling effect that limited its sensitivity for detecting individual differences, and consequently, it was not used as a dependent variable alongside silent reading speed in the present analyses. It is thus possible that reading comprehension scores obtained during the experimental task may not have been sufficiently sensitive to detect unforeseen age differences in the degree of online cognitive effort exerted during silent passage reading. Nevertheless, the comprehension questions fulfilled their intended purpose of ensuring participants’ minimum level of engagement with the passages beyond word-level identification or reading solely for speed. Accordingly, the ceiling effect observed in the experimental comprehension questions reflects their limited discriminatory power rather than an absence of meaningful comprehension during the eye-tracking task. Future work will extend current results by including passages of varying difficulty and a more sophisticated assessment of reading comprehension, allowing a more systematic examination of whether the eye-movement predictors identified here explain not only reading speed but also the comprehension-based reading performance of children.

Fourth, the use of relatively short passages may limit the ecological validity of the results, especially for older children who increasingly encounter longer texts in their curricula. Short passages were chosen to balance ecological considerations with methodological requirements. Passages were sufficiently long to afford a sufficient number of fixation observations per passage, to allow reliable estimation of Ex-Gaussian parameters, without imposing the sustained attentional demands and reading-related fatigue that longer passages would place on children in this age range (especially the younger ones). Additionally, short passages are more easily administered within the constraints of testing within the school environment. In view of this limitation, generalizability of the present findings to longer and more extended reading activities remains to be established.

A final potential concern is that silent reading speed and several eye-movement parameters, such as forward fixations per word and mean fixation duration, are partially mathematically dependent, since they were derived from the same task. Accordingly, the regression coefficients reported here should be interpreted as describing associations within a common reading process rather than evidence of causal mechanisms. This concern applies less directly to distributional descriptors such as the Ex-Gaussian parameter τ, which characterizes the shape of the fixation-duration distribution rather than contributing directly to the calculation of reading speed. Nevertheless, because all measures were obtained from the same reading task, even these associations should be interpreted cautiously. Future longitudinal and experimental studies will be needed to determine whether the present oculomotor measures prospectively predict improvements in reading performance or change in response to intervention.

## 5. Conclusions

In conclusion, the present study highlights that children’s silent reading speed measures are associated with changes in their oculomotor behavior across primary school years. Among the examined eye-movement measures, forward fixation number was the most robust predictor of silent reading speed, while the Ex-Gaussian parameter τ explained additional variance beyond mean fixation duration, highlighting the value of distributional analyses of fixation behavior. Although the cross-sectional design precludes conclusions regarding the direction or causality of these associations, the current findings add significantly to the growing body of evidence suggesting that oculomotor behavior is a robust correlate of reading fluency. Replication in larger and more geographically diverse samples would strengthen the generalizability of the present findings, while future longitudinal studies are expected to clarify the mechanisms underlying the current associations.

## Figures and Tables

**Table 1:** Demographic, vision, language and cognitive characteristics of the sample by grade (G3–G6).

	Mean (SD)				*p* Value					
Measure	G3	G4	G5	G6	G3–4	G3–5	G3–6	G4–5	G4–6	G5–6
Girls (N)	28	30	26	29	0.78	0.85	0.98	0.93	0.75	0.83
Age (years)	8.7 (0.3)	9.7 (0.3)	10.8 (0.4)	11.7 (0.4)	**<0.001**	**<0.001**	**<0.001**	**<0.001**	**<0.001**	**<0.001**
Visual Acuity ^1^	−0.08 (0.08)	−0.08 (0.09)	−0.07 (0.01)	−0.12 (0.07)	0.81	0.49	0.01	0.69	0.01	**<0.001**
Reading Acuity ^2^	1.10 (0.14)	1.08 (0.02)	1.17 (0.02)	1.19 (0.11)	0.38	0.01	<0.001	0.73	0.84	0.34
Receptive Vocabulary ^3^	54 (9)	50 (7)	49 (9)	52 (10)	0.01	0.01	0.18	0.73	0.36	0.28
ORF, wpm ^4^	79 (22)	92 (25)	108 (27)	124 (28)	0.01	**<0.001**	**<0.001**	<0.01	**<0.001**	0.01
ORF, %ile ^4^	57 (26)	54 (28)	55 (30)	51 (28)	0.48	0.67	0.21	0.82	0.60	0.47
Text RC %ile ^5^	89.1 (9.9)	81.2 (14.0)	66.9 (22.2)	66.9 (23.2)	**0.002**	**<0.001**	**<0.001**	**<0.001**	**<0.001**	0.99
RAN Digits ^6^	116 (23)	123 (25)	139 (26)	142 (25)	0.19	**<0.001**	**<0.001**	**0.00**	**<0.001**	0.57
RAN Objects ^6^	64.14 (12)	70 (14)	76 (12)	79 (17)	0.03	**<0.001**	**<0.001**	0.02	0.01	0.45
Symbol Search ^7^	12.5 (2.5)	11.5 (2.0)	11.6 (2.9)	11.7 (2.4)	0.03	0.14	0.07	0.72	0.81	0.89
Coding ^7^	9.6 (2.4)	10.4 (3.1)	9.5 (2.6)	10.0 (2.5)	0.26	0.90	0.42	0.23	0.67	0.37

^1^ ETDRS charts; logMAR. ^2^ Colenbrander reading charts; logMAR. ^3^ LEXiS; scaled score. ^4^ ORF = Oral Reading Fluency, Test-A Passage Reading subtest; wpm = words per minute. ^5^ Text-level Reading Comprehension (grade-adjusted percentile rank based on the standardized Passage Reading subtest from the TEST-A battery). ^6^ RAN = Rapid Automatized Naming; items per minute. ^7^ WISC-V subtests (scaled scores). Note: *p* values are for independent sample *t*-tests. Statistically significant comparisons are noted in bold (at Bonferroni-adjusted *p* < 0.05/6 = 0.008).

**Table 2:** Descriptive statistics for passage-level silent reading speed and concurrent eye-movement variables.

	Mean (SD)				*p* Value					
Measure	G3	G4	G5	G6	G3–4	G3–5	G3–6	G4–5	G4–6	G5–6
N	49	50	44	51						
Silent Reading Speed (wpm)	111 (55)	127 (47)	155 (57)	166 (44)	0.14	**<0.001**	**<0.001**	0.01	**<0.001**	0.28
Saccade Amplitude (letters)	4.9 (1.9)	5.3 (1.7)	6.6 (2.1)	6.8 (1.7)	0.32	**<0.001**	**<0.001**	0.00	**<0.001**	0.71
Forward Fixations/word	1.33 (0.51)	1.20 (0.42)	0.96 (0.28)	0.91 (0.22)	0.15	**<0.001**	**<0.001**	0.00	**<0.001**	0.33
Regression (%)	29.0 (5.9)	27.3 (6.4)	29.1 (6.9)	28.6 (7.9)	0.19	0.92	0.82	0.20	0.37	0.77
Fixation Duration (ms)	293 (49)	279 (47)	256 (41)	242 (28)	0.14	**<0.001**	**<0.001**	0.01	**<0.001**	0.06
Ex-Gaussian μ (ms)	153 (18)	154 (20)	145 (14)	147 (18)	0.78	0.04	0.12	0.02	0.08	0.64
Ex-Gaussian τ (ms)	139 (45)	124 (43)	109 (36)	94 (26)	0.09	0.00	**<0.001**	0.07	**<0.001**	0.02
Ex-Gaussian σ (ms)	33 (9)	33 (11)	29 (6)	32 (8)	0.90	0.04	0.60	0.06	0.55	0.11
Blinks/min	4.9 (5.7)	5.3 (5.1)	5.3 (4.2)	5.6 (4.5)	0.44	0.39	0.45	0.56	0.75	0.92
Comprehension (%)	90.3 (9.9)	92.4 (8.7)	94.7 (5.8)	94.8 (6.2)	0.27	0.03	0.02	0.31	0.25	0.92

G3–G6 = Grades 3 to 6; wpm = words per minute. Unadjusted *p* values are for independent sample *t*-tests for all measures, with the exception of blink rate and comprehension accuracy, where the Kruskal–Wallis test was used. Statistically significant comparisons are noted in bold (at Bonferroni-adjusted *p* < 0.05/6 = 0.008).

**Table 3:** Hierarchical regression analysis, predicting silent reading speed from demographics, cognitive-speed abilities, and eye-movement parameters (N = 194).

Predictor	B	SE	β	t	*p*	95% CI	ΔR^2^ (Variable)
Step 1: Demographic, cognitive-speed, and language predictors							
Age (years)	10.354	3.315	0.225	3.12	0.002	[3.86, 16.85]	0.034
Female Gender	−5.585	7.259	−0.050	−0.77	0.44	[−19.81, 8.64]	0.002
RAN Digits	0.564	0.150	0.277	3.76	<0.001	[0.27, 0.86]	0.049
RAN Objects	0.170	0.279	0.046	0.61	0.54	[−0.38, 0.72]	0.001
Coding	0.550	1.466	0.027	0.38	0.71	[−2.32, 3.42]	<0.001
Symbol Search	2.821	1.494	0.128	1.89	0.06	[−0.11, 5.75]	0.012
Receptive Vocabulary	1.765	0.520	0.215	3.40	0.001	[0.75, 2.78]	0.040
*Adj. R*^2^ = 0.330, *F* (7, 186) = 14.60, *p* < 0.001							
Step 2: +Eye-movement predictors							
Age (years)	0.108	1.893	0.002	0.06	0.95	[−3.60, 3.82]	<0.001
Female Gender	−6.387	3.977	−0.057	−1.61	0.56	[−14.18, 1.41]	0.003
RAN Digits	0.052	0.086	0.026	0.61	0.54	[−0.12, 0.22]	<0.001
RAN Objects	−0.073	0.153	−0.020	−0.48	0.64	[−0.37, 0.23]	<0.001
Coding	0.036	0.809	0.002	0.04	0.96	[−1.55, 1.62]	<0.001
Symbol Search	1.162	0.825	0.053	1.41	0.16	[−0.46, 2.78]	0.002
Receptive Vocabulary	0.862	0.294	0.105	2.93	0.004	[0.29, 1.44]	0.009
Forward fixations per word	−83.485	6.005	−0.622	−13.90	<0.001	[−95.25, −71.71]	0.200
Regression %	−1.650	0.273	−0.205	−6.04	<0.001	[−2.19, −1.12]	0.038
Ex-Gaussian τ (ms)	−0.338	0.060	−0.256	−5.62	<0.001	[−0.46, −0.22]	0.033
*Adj. R*^2^ = 0.800, Δ*R*^2^ (step) = 0.456, Δ*F* (3, 183) = 147.03, *p* < 0.001							

B = unstandardized regression coefficient; SE = standard error of B; β = standardized regression coefficient; 95% CI = 95% confidence interval for B; ΔR^2^ (variable) = squared semi-partial correlation, representing the amount of explained variance added by each predictor, beyond all other predictors in the model; ΔR^2^ (step) = total increase in explained variance when all three eye-movement predictors were entered together in Step 2, relative to the Step 1 model. RAN: Rapid Automatized Naming. Coding and Symbol Search are WISC-V subtests. All models were estimated using the Enter method. For grade-level multiple regression models, see Table A2 in Appendix A.

**Figure A1:**
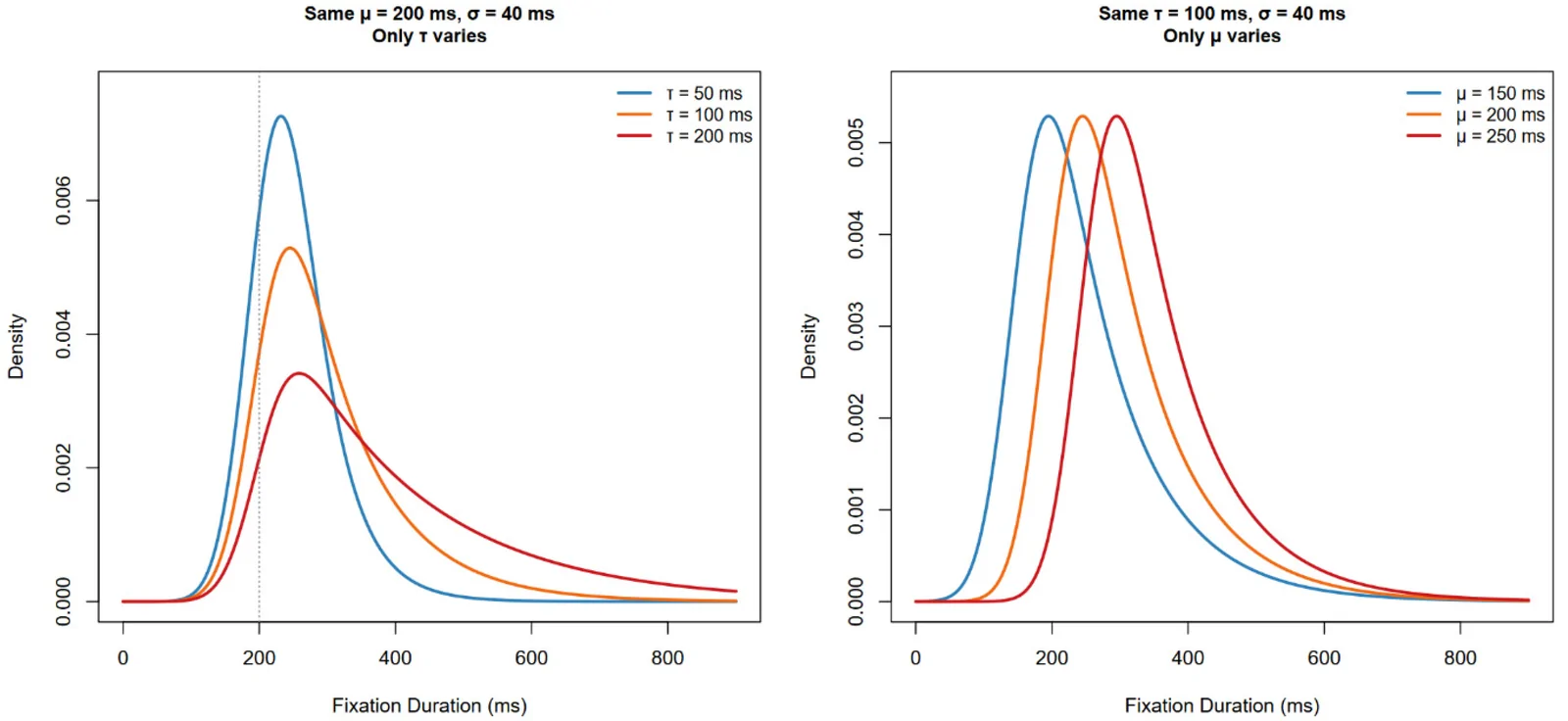
Simulated Ex-Gaussian distributions illustrate the distinct effects of τ and μ on distribution shape. The dashed line corresponds to the Ex-Gaussian parameter μ, kept stable at 200 ms.

**Figure A2:**
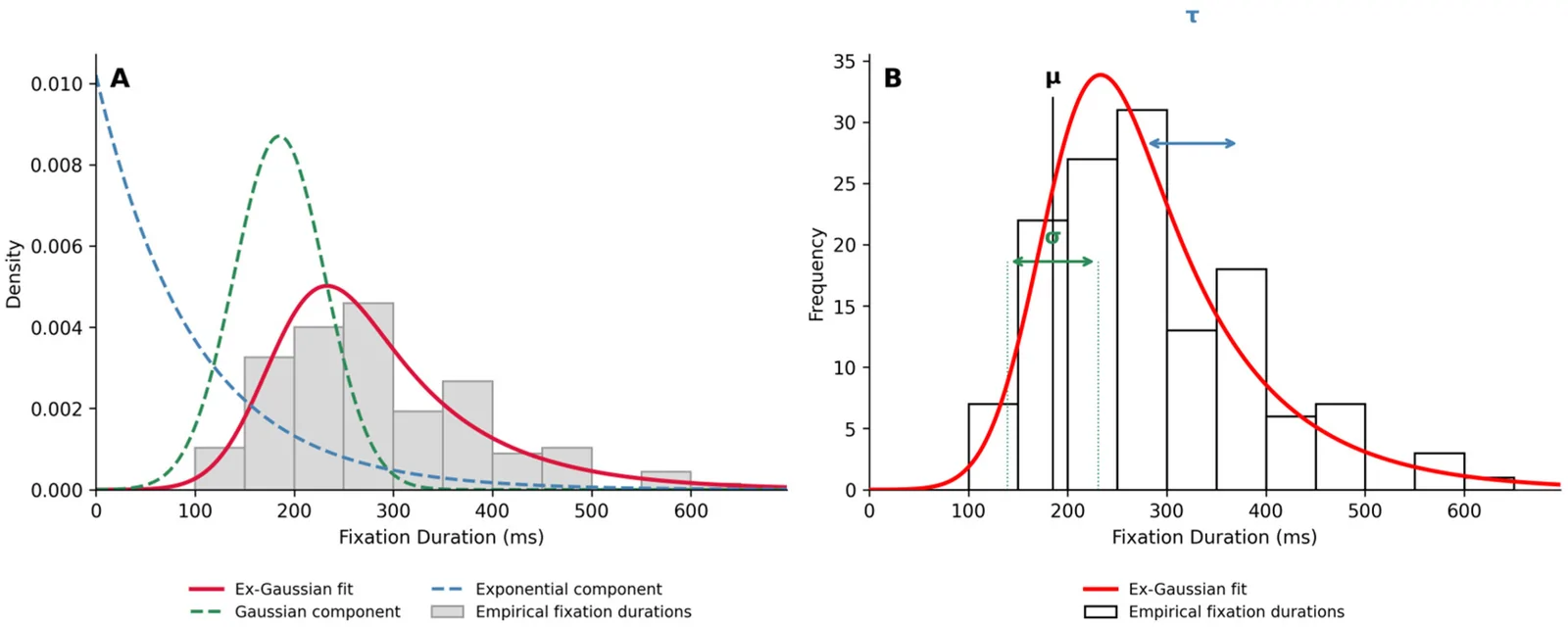
(**A**) Histogram of fixation durations for a single passage as read by one participant attending Grade 3; the fitted Ex-Gaussian distribution (red) is decomposed into its Gaussian component (green dashed; providing parameters μ and σ) and the exponential component (blue dashed; providing parameter τ). (**B**) The same passage’s fitted Ex-Gaussian distribution shown as a single curve, with μ marking the center of the Gaussian component, σ (green) indicating the width of the Gaussian component (μ ± σ), and τ (blue) indicating the characteristic decay length of the exponential tail. μ = 185 ms, σ = 46 ms, τ = 98 ms.

## Data Availability

The data presented in this study are available on request from the corresponding author due to ethical and privacy restrictions concerning child participant data.

## Abbreviations

The following abbreviations are used in this manuscript:

bpm	Blinks per minute
fpw	Fixations per word
G3–G6	Grades 3–6
logMAR	logarithm of the Minimum Angle of Resolution
ORF	Oral reading fluency
RAN	Rapid Automatized Naming task
WISC-V	Wechsler Intelligence Scale for Children—Fifth Edition
wpm	Words per minute

## Appendix A

To illustrate how τ shapes the right tail of the fixation-duration distribution, three Ex-Gaussian distributions were simulated, while holding μ and σ constant (at 200 ms and 40 ms, respectively) and varying τ (50, 100, and 200 ms). As shown in Figure A1 (left panel), increasing τ progressively produces a heavier right tail, substantially increasing the probability of prolonged fixations while leaving the modal region of the distribution largely unchanged. To further illustrate the distinct roles of μ and τ, simulated Ex-Gaussian distributions were created while holding τ and σ constant and varying only μ. Although increases in μ shift the central location of the distribution, they have a much smaller effect on the probability of prolonged fixations (>500 ms). These simulations illustrate that, although both parameters influence the overall distribution, τ exerts a substantially stronger influence on the prevalence of unusually long fixations (reflected in a heavily skewed right tail), whereas μ primarily shifts the central tendency, with comparatively modest effects on the upper tail. This distinction supports the interpretation of τ as an index of relatively infrequent processing delays that are not fully captured by mean fixation duration, thereby providing complementary information about individual differences in reading efficiency.

An example of a fitted Ex-Gaussian distribution for the fixation duration data recorded during reading a single passage in one participant is shown in Figure A2.

**Table A1:** Ex-Gaussian vs. Gaussian-only model fit (BIC) by grade.

Grade	N Passages	Median BIC (Ex-Gaussian)	Median BIC (Gaussian-Only)	Mean ΔBIC
3	231	1685	1724	74
4	233	1402	1463	59
5	209	1319	1352	43
6	248	1171	1183	38

Note. ΔBIC = Gaussian BIC minus Ex-Gaussian-only BIC; positive values favor the Ex-Gaussian model. All grade-level values exceed the conventional threshold of 10 points considered “very strong” evidence [62] (Kass & Raftery, 1995). There is a notable reduction of ΔBIC with grade, although the Ex-Gaussian model remains strongly favored over the Gaussian fit at every grade.

**Table A2:** Multiple regression models predicting silent reading speed by eye-movement parameters, conducted separately in Grades 3, 4, 5, and 6.

Predictor	B	SE	β	t	*p*	95% CI	ΔR^2^ (Variable)
*Grade 3* (N = 49, *Adj. R*^2^ = 0.759)							
Forward fixations per word	−68.603	9.561	−0.631	−7.18	<0.001	[−87.34, −49.86]	0.258
Regression %	−2.095	0.656	−0.226	−3.19	0.003	[−3.38, −0.81]	0.051
Ex-Gaussian τ (ms)	−0.369	0.107	−0.303	−3.44	0.001	[−0.58, −0.16]	0.059
*Grade 4* (N= 50, *Adj. R*^2^ = 0.861)							
Forward fixations per word	−79.677	8.746	−0.706	−9.11	<0.001	[−96.82, −62.54]	0.235
Regression %	−1.809	0.401	−0.245	−4.51	<0.001	[−2.60, −1.02]	0.058
Ex-Gaussian τ (ms)	−0.210	0.085	−0.191	−2.47	0.017	[−0.38, −0.04]	0.017
*Grade 5* (N= 44, *Adj. R*^2^ = 0.824)							
Forward fixations per word	−143.871	14.059	−0.700	−10.23	<0.001	[−171.43, −116.32]	0.429
Regression %	−2.189	0.528	−0.265	−4.15	<0.001	[−3.22, −1.15]	0.070
Ex-Gaussian τ (ms)	−0.521	0.107	−0.332	−4.85	<0.001	[−0.73, −0.31]	0.096
*Grade 6* (N= 51, *Adj. R*^2^ = 0.854)							
Forward fixations per word	−147.391	12.338	−0.728	−11.95	<0.001	[−171.57, −123.21]	0.417
Regression %	−1.391	0.308	−0.252	−4.52	<0.001	[−2.00, −0.79]	0.060
Ex-Gaussian τ (ms)	−0.543	0.103	−0.321	−5.27	<0.001	[−0.74, −0.34]	0.081

Note. Each model predicts silent reading speed from forward fixations per word, regression percentage (%), and Ex-Gaussian parameter τ simultaneously (Enter method). B = unstandardized coefficient; SE = standard error of B; β = standardized coefficient; 95% CI = confidence interval for B; ΔR^2^ (variable) = squared semi-partial correlation, representing the amount of explained variance uniquely added by each individual predictor, beyond all other predictors in the model.
